# Marginal integrity of low-shrinkage and methacrylate-based composite resins: Effect of three different hemostatic agents

**DOI:** 10.4317/jced.52782

**Published:** 2016-04-01

**Authors:** Maryam Khoroushi, Farinaz Shirban, Mahsa Sahraneshin-Samani

**Affiliations:** 1 DDS, MS. Professor, Dental Materials Research Center, Department of Operative Dentistry, Isfahan University of Medical Sciences, Isfahan, Iran; 2 DDS, MS. Assistant Professor, Torabinejad Dental Research Center, Department of Orthodontics, School of Dentistry, Isfahan University of Medical Sciences, Isfahan, Iran; 3 DDS, MS. Assistant Professor, Department of Operative Dentistry, School of Dentistry, Shahrekord University of Medical Sciences, Shahrekord, Iran

## Abstract

**Background:**

Moisture control is very important in restorative procedures in dentistry. Use of hemostatic agents helps control moisture; however, they might result in changes on enamel and dentin surfaces, affecting composite resin bond quality. The aim of this in vitro study was to evaluate the marginal microleakage of two different composite resins with the use of three different hemostatic agents.

**Material and Methods:**

Standardized Class V cavities were prepared on the buccal and lingual surfaces of 48 premolars with cervical margins 1 mm apical to the cementoenamel junction (CEJ). The samples were randomly divided into 8 groups. In groups 1 to 4, an etch-and-rinse adhesive (Adper Single Bond) was applied as the bonding system, followed by exposure to different hemostatic agent: group 1: no hemostatic agent (control); group 2: ViscoStat; group 3: ViscoStat Clear; and group 4: trichloracetic acid, as hemostatic agents. The cavities were restored with Z-250 composite resin. In group 5 to 8 Silorane System Adhesive (Filtek P90 Adhesive) was applied as a bonding agent, followed by exposure to different hemostatic agents in a manner similar to that in groups 1to 4. The cavities were restored with Filtek P90, a low-shrinkage composite resin. The samples in each group were evaluated for dye penetration under a stereomicroscope at ×36 after 24 hours and a 500-round thermocycling procedure at enamel and dentin margins. Statistical analysis was carried out using Kruskal-Wallis and Mann-Whitney tests (α=0.05).

**Results:**

Z-250 composite resin exhibited significantly higher dentin microleakage scores compared to Filtek P90 (*P* = 0.004). Trichloracetic acid increased dentin microleakage with Filtek P90 (*P*=0.033).

**Conclusions:**

Under the limitations of this in vitro study, application of hemostatic agents did not affect microleakage of the two tested composite resins except for trichloracetic acid that increased marginal microleakage when used with Filtek P90.

** Key words:**Composite resin, dental leakage, hemostatics, silorane system adhesive.

## Introduction

The past decade has witnessed great advances in bonding techniques, technologies and applications in the dental field, resulting in adhesion proper bond between resins and tooth structures. To achieve a durable high-performance composite resin restoration, it is necessary to provide proper isolation, especially near or at gingival margins. Contamination of the areas to be bonded can have a detrimental effect on the longevity of the restoration, hampering its clinical performance and success (1). Contaminated cavities impair visualization and accessibility, resulting in microleakage, which in turn can give rise to tooth hypersensitivity, pulpal irritation, tooth discoloration, recurrent caries, less durable restorations and eventually clinical failure (2).

Silorane-based composite resins are formed through reactions between oxirane and siloxane molecules; this type of composite resin has a ring-opening polymerization reaction, resulting in minimal polymerization shrinkage. The methacrylate-based composite resins exhibit 2.3-3% of shrinkage in their volume; however, silorane-based composite resins have been reported to exhibit volumetric shrinkage of approximately 0.9%, leading to less stress on the cavity walls (3). Therefore, Filtek Silorane is supplied with a two-step self-etch adhesive, referred to as Silorane System Adhesive (SSA), compatible with the highly hydrophobic silorane matrix. The self-etch primer in the SSA cannot remove the smear layer within the dentinal tubules due to its pH value of 2.7 and is classified as an ultra-mild etchant (4).

In recent years, use of hemostatic agents to control gingival bleeding and reduce sulcular fluid has been advocated. They have been presented in various formulations with different mechanisms of action, such as aluminum chloride, ferric sulfate compounds, iron solution, aluminum and potassium sulfate and 0.1% epinephrine (5). Trichloracetic acid (TCA), Viscostat (VS) and Viscostat Clear (VSC) hemostatic agents provide effective isolation from sulcular fluid, blood and saliva. VS is a proprietary name for a 20% ferric sulfate solution while VSC consists of a 25% aluminum chloride solution. Hemostasis with these solutions is mediated through coagulum plugs pushed into capillary fenestrations (6,7). Recent studies have shown that majority of these hemostatic agents have acidic properties (pH = 0.7-3) and hydrophilic characteristics that can contaminate all the stages of bonding procedures (5,8). The smear layer has been removed, affecting the hybrid layer quality after application of self-etch or etch-and-rinse adhesives (9,10). Hemostatic agents might change the dentin surface morphology (11). Previous studies have shown that hemostatic agents can affect the bond strength of adhesive resins (6,7,10,12-14). The bond strength of self-etching adhesive systems is affected more negatively than that of etch-and-rinse systems (11). Considering the acidity of these agents, contamination of dental substrates with these materials might have a detrimental effect on the bond strength (7). Most hemostatic agents are soluble in water; therefore, vigorous rinsing with water spray has been advocated before any bonding procedure (15).

Little is known about the local effects of hemostatic agents on the enamel and dentin substrates upon hybridization (11). There is insufficient knowledge available on the effect of TCA, VS and VSC hemostatic agents on microleakage of composite resin restorations with the application of different adhesive resins systems, including SSA. This study was undertaken to evaluate the micro-leakage of one etch-and-rinse and one SSA on human dentin contaminated with three hemostatic agents. The null hypothesis tested was that these hemostatic agents do not affect gingival margin microleakage.

## Material and Methods

Forty-eight extracted non-carious human premolars, stored in 0.2% thymol solution at 4°C, were evaluated in the present study. Twenty-four hours before the study procedures, the teeth were removed from the thymol solution and stored in distilled water at 37°C before being prepared. Class V cavities (2 mm in depth, 3 mm mesiodistally, 2 mm occluso-gingivally) were prepared at the cementoenamel junction on both buccal and lingual surfaces, with the occlusal and gingival margins located in enamel and dentin, respectively. The cavities were prepared using a diamond bur (D & Z, Hilzingen, Germany) in a high-speed handpiece under water spray. Before tooth preparation the dimension of each cavity was drawn on each tooth using standard templates and the depth of prepared cavity was measured by a periodontal probe. Subsequent to preparation, all the specimens were randomly assigned to eight groups according to the contamination material applied, as follows:

• Group 1 (control): The dentin surface was air-dried to remove excess moisture. Then the dentin surfaces were etched with 37.5% phosphoric acid (Kerr, OA, USA) for 15 seconds, rinsed for 30 seconds and blot-dried. Then Adper Single Bond (3M ESPE, MN, USA) etch-and-rinse adhesive was applied on the cavity walls according to manufacturer’s instructions. LED light-curing system (Demi LED Light-curing System, Kerr Corp, OA, USA) was used for light-curing procedures at an intensity of 1000 mW/cm2 perpendicular to the surface.

A3 shade of Z250 composite resin (3M ESPE, MN, USA) was used to restore the cavities using the incremental technique (two 1-mm layers). Each layer was cured for 20 seconds.

• Group 2: All the procedures were the same as those in group 1 except for the fact that before the application of Single Bond adhesive resin, VS (Ferric Sulfate, Ultradent, USA) hemostatic agent was applied for 2 minutes at margins using a mini-brush, followed by rinsing for 30 seconds and drying with air spray.

• Group 3: All the procedures were the same as those in group 2 except for the fact that VSC (Aluminum Chloride, Ultradent, USA) was applied as a hemostatic agent.

• Group 4: All the procedures were the same as those in groups 2 and 3 except that TCA was applied as a hemostatic agent.

• Group 5 (control): Dentin surface was air-dried to remove excess moisture, Then Primer of “Filtek P90 Adhesive” (3M ESPE, USA) was applied to the cavity by a microbrush and was distributed with gentle air pressure and light-cured with LED (Demi LED light curing system, Kerr Corp, USA) at 1100-1200 mW/cm2 light-curing unit for 10 seconds; then bonding of “Filtek P90 Adhesive” (3M ESPE) was applied and distributed with gentle air pressure and light-cured for 10 seconds. Filtek P90 composite resin (Filtek P90, shade A3, 3M ESPE) was used to restore the cavities using the incremental technique (two 1-mm layers). Each layer was cured for 20 seconds.

• Group 6: All the procedures were the same as those in group 5 except for the fact that before the application of Filtek P90 Adhesive, VS hemostatic agent was applied.

• Group 7: All the procedures were the same as those in group 6 except that VSC hemostatic agent was used.

• Group 8: All the procedures were the same as those in groups 6 and 7 except for the fact that TCA hemostatic agent was applied.

-Preparation of samples for dye penetration

After the restorative procedures, the samples were stored in distilled water for 24 hours. Subsequently, the samples underwent a 500-cycle thermocycling procedure at 5°C/55°C±2° with a dwell time of 30 seconds. The apical foramina of the teeth were occluded with wax and then the entire tooth surface was painted with two coats of air-resistant varnish (nail polish) to within 1 mm of the restoration margins. The teeth were immersed in 2% basic fuchsin dye for 24 hours. Each tooth was sectioned in the bucco-lingual direction through the center of the bulk of Cl V restorations and evaluated under a stereomicroscope at ×36.

The following scoring criteria was followed for dye penetration at composite resin‒tooth interface:

• 0 = No dye penetration

• 1 = Dye penetration up to, but not beyond, ½ of the gingival wall

• 2 = Dye penetration up to, but not contacting, the axial wall

• 3= Dye penetration along the axial wall (16)

Data were analyzed using the Kruskal-Wallis and Mann-Whitney test, *P* value <0.05 was considered statistically significant.

## Results

Kruskal-Wallis test did not show any significant differences in the means of enamel microleakage between the eight groups evaluated (*P*=0.187); however, dentin microleakage was statistically significant between the different study groups (*P*<0.05). According to Mann-Whitney test differences in the dentin microleakage of the two adhesives under study were significant; the samples in group 1 exhibited significantly higher microleakage scores compared to group 5 (*P*=0.004). In addition, the samples in group 5 exhibited significantly lower microleakage scores compared to group 8 (*P*=0.033). Tables  Table 1 and  Table 2 summarize the micro-leakage scores of all the groups.

**Table 1:**
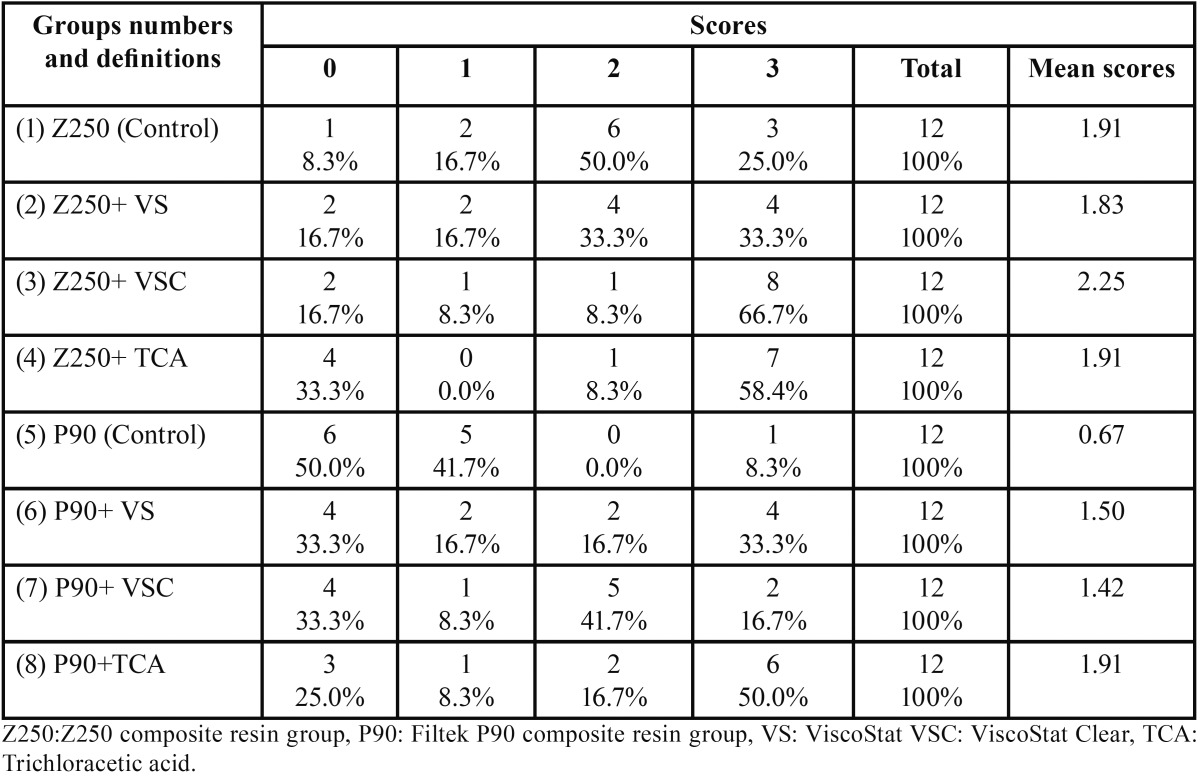
Microleakage distribution in dentin margins in the study groups.

**Table 2:**
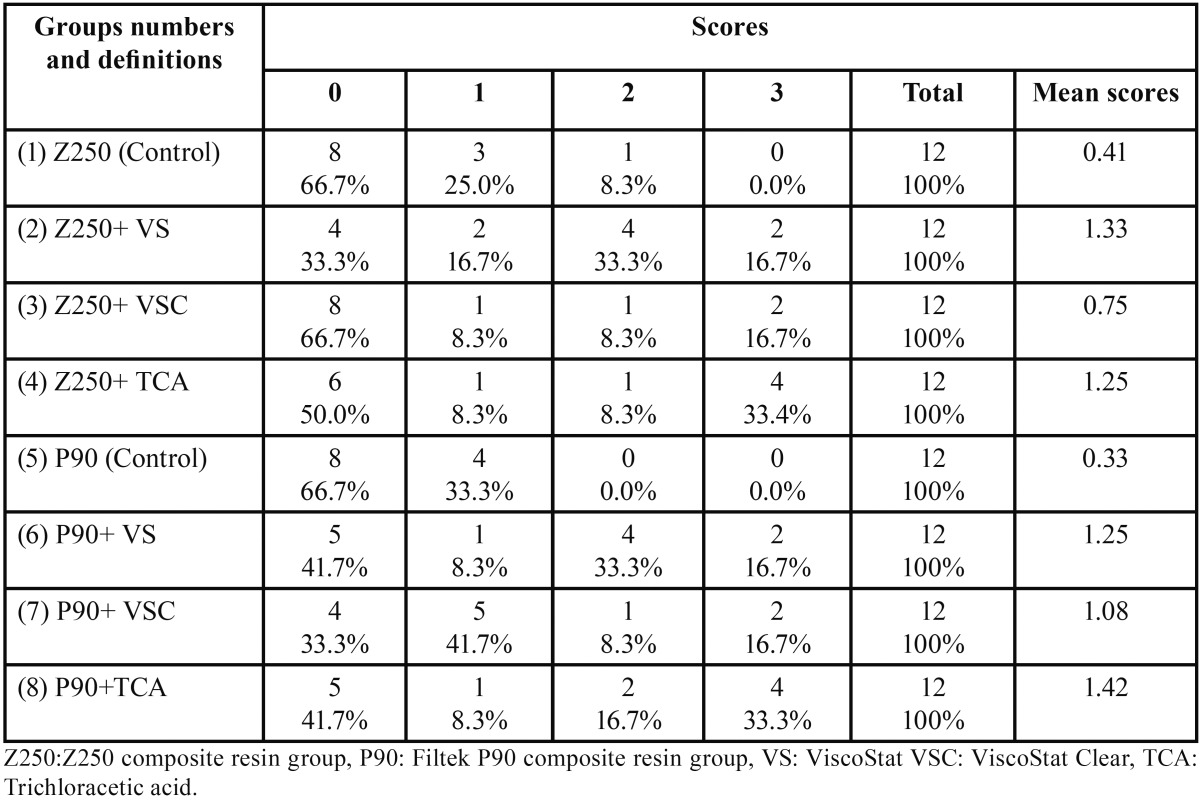
Microleakage distribution in enamel margins in the study groups.

## Discussion

In this study the effect of contamination of dentin and enamel with three different hemostatic agents (TCA, VS and VSC) on the marginal microleakage of an etch-and-rinse adhesive system and SSA were evaluated. All the groups exhibited some dye penetration at tooth-restoration interfaces, which can be attributed to dimensional changes due to polymerization shrinkage of restorative resins, and the differences in thermal expansion coefficients between the teeth and restorative materials. Such changes give rise to internal forces in composite resin materials, leading to gap formation at tooth-restoration interfaces and microleakage (17).

The results of the present study showed that application of hemostatic agents (except for TCA in Filtek P90 Adhesive at dentinal margin) did not result in statistically significant differences in microleakage of either methacrylate-based or silorane-based resin composites. In another study contamination with hemostatic agents did not affect microleakage when a two-step self-etching adhesive system was used (18). In a study by Kumar *et al.* when the cavities were exposed to VS, Single Bond exhibited significantly higher microleakage at gingival margins compared to the controls (2). In the study above the cavities were first etched with phosphoric acid and then VS was applied to cavity walls; moreover, contamination time was 10 seconds versus 2 minutes in this study (2).

In the present study Single Bond exhibited higher microleakage at dentin margins compared to Filtek P90 Adhesive (SSA). According to the literature, etch-and-rinse adhesives completely remove the smear layer and peritubular dentin subsequent to phosphoric acid etching, increasing fluid movement across the resin‒dentin interface. Conversely, self-etching systems result in less fluid movement due to a less aggressive etching pattern, resulting in superior dentin sealing compared to the etch-and-rinse system (19). In addition, silorane-based composite resins used with SSA exhibit a unique and low-shrinkage matrix with fillers in the adhesive system. This filler-containing adhesive agent results in a relatively strong hybrid layer with hydrolytic stability. Furthermore, in SSA the primer and bonding component are light-cured separately. In order to match with the hydrophobic silorane composite resin, the bonding agent contains hydrophobic bifunctional monomers in its composition, explaining why this two-step procedure can improve the quality of tooth-composite resin interfaces (3). Light polymerization reaction of silorane is cationic and exhibits greater affinity for oxygen compared to free radical polymerization and does not result in an air-inhibited layer. Therefore, not only polymerization shrinkage decreases, but also due to this effect, the degree of conversion increases in silorane adhesive component (20).

This study showed that contamination of dentin with TCA decreases the marginal integrity of Filtek P90 adhesive, compared to the control group. In addition, subsequent to the contamination of dentin surface and application of etch-and-rinse adhesive (groups 2 to 4) marginal microleakage did not increase. TCA is an acid (pH≤1.0.) A microscopic evaluation showed an over-etching pattern that is more prominent with 50% TCA. The etched surface of dentin may be too deep to be penetrated by the limited diffusion of adhesive, especially with the use of a viscous self-etch SSA (21). On the other hand, pH of self-etch adhesive systems determines their ability to interact with underlying tissues. The lower the pH value of the adhesive system, the more aggressive it is, resulting in complete removal of the smear layer (22). The adhesive system used in this study has an ultra-mild aggressive potential (pH>2.0), making it more sensitive to the presence of any substance on tooth surface. The phosphoric acid in chemical composition of etch-and-rinse adhesives helps remove most of the contaminants from the dentin surface before the application of the adhesive resin (8). The aggressive etching induced by phosphoric acid with pH≤0.5 might remove all the contaminants on the dentin surface (11). This was supported by the results of the energy-dispersive spectrometer analysis, which showed a similar volume of aluminum remaining on the surfaces of normal and contaminated dentin after etching by phosphoric acid (13).

The exposure of prepared surfaces of dentin to gingival retraction fluids resulted in changes in its morphology, decreasing the dentin’s susceptibility to acid-etching. Acidic gingival retraction fluids might have dissolved the dentin apatite crystallites, resulting in a large increase in local concentrations of mono-, di- and trivalent phosphate. This, along with the available calcium, formed a fine amorphous layer of calcium phosphates along with insoluble aluminum phosphates, which might account for the presence of granular precipitates in the VSC observed after 5 minutes of exposure. The energy-dispersive X-ray analysis supports the view that these precipitates might result from the interaction between insoluble calcium and aluminum phosphates. The ferric phosphate that might have formed with VS, being more soluble than aluminum phosphate, might have been dissolved and washed away from the surface by water (23). As a result, subsequent to the contamination of cavities with VS in etch-and-rinse adhesive system, marginal leakage decreased compared to VSC, although this was not statistically significant.

Based on previous studies, the most detrimental effects of VS on bond strength and marginal seal occur when all-in-one adhesives are used. The bonding effectiveness depends on the smear layer; however, the quality of bonding for etch-and-rinse adhesives is least affected (24).

## Conclusions

Within the limitations of this in vitro study, contact of the hemostatic agents (VS, VSC, TCA) with prepared cavity walls did not affect marginal integrity of two composite resins evaluated in this study, except for trichloracetic acid when it was used with a low-shrinkage composite resin.
